# Inhibition of HCV 3a core gene through Silymarin and its fractions

**DOI:** 10.1186/1743-422X-8-153

**Published:** 2011-04-01

**Authors:** Usman Ali Ashfaq, Tariq Javed, Sidra Rehman, Zafar Nawaz, Sheikh Riazuddin

**Affiliations:** 1 Division of Molecular Medicine, National Centre of Excellence in Molecular Biology, University of the Punjab, Lahore, Pakistan; 2 Braman Family Breast Cancer Institute, University of Miami, USA; 3 Allama Iqbal Medical College, University of Health sciences, Lahore

## Abstract

Hepatitis C is a major health problem affecting 270 million individuals in world including Pakistan. Current treatment regimen, interferon alpha and ribavirin only cure half of patients due to side effects and high cost.

**Results:**

In the present study *Silybum marianum *(Milk thistle) seeds were collected, extracted and analyzed against HCV 3a core gene by transiently transfecting the liver cells with HCV core plasmid. Our results demonstrated that Silymarin (SM) dose dependently inhibit the expression or function of HCV core gene at a non toxic concentration while the GAPDH remained constant. To identify the active ingredient, SM was fractioned by thin layer chromatography (TLC), column chromatography and HPLC. Purified fractions were tested for HCV core gene and western blotting results showed that two factions of SM (S1 and S2) inhibit HCV 3a core expression or function in liver cells

**Conclusion:**

Our results suggest SM and its fractions (S1 and S2) inhibit HCV core gene of 3a genotype and combination of SM and its fractions with interferon will be a better option to treat HCV infection

## Background

HCV is a leading cause of variety of liver diseases varying from asymptomatic condition to hepatocellular carcinoma. HCV affects 270 million people worldwide and 10-15 million people are the carriers of HCV in Pakistan [1]. It was estimated by the World Health Organization in 2004 that the annual deaths due to liver cancer caused by HCV and cirrhosis were 308,000 and 785,000, respectively [2]. HCV is a blood-borne pathogen, which transmitted through parenteral exposure to contaminated blood or body fluids [3]. Factors most strongly associated with HCV infection are blood transfusion (56%), alcohol consumption (44%) and intravenous drug abuse (31%) [3,4]. Some other risk factors include use of inadequately sterilized medical equipment, high-risk sexual behaviors, and social or cultural practices such as body piercing, circumcision, and tattooing [5,6].

Pegylated interferon (PEG-IFN) plus Ribavirin therapy is the current treatment for the patient with chronic hepatitis C. The main goal of therapy is to achieve a sustained virological response currently defined as undetectable HCV-RNA in peripheral blood determined with the most sensitive polymerase chain reaction technique 24 weeks after the end of treatment. This goal is practically equivalent with eradication of HCV infection and cure of the underlying HCV induced liver disease. This therapy cure only half number of patients due to side effects, resistance and high cost [7-10]. Hence, there is a need to develop anti-HCV agents, both from herbal sources and synthetic chemistry which are less toxic, more efficacious and cost-effective. The present study was undertaken to study the effect of SM and its fraction against HCV 3a core gene in liver cells. We report here that SM and its fractions effectively inhibited Core gene RNA and protein expression in a dose-dependant manner in Huh-7 cells.

## Materials and methods

### Extraction of *Silybum marianum*

The seeds of *Silybum marianum *were collected from Township market and SM was extracted as described by Polyak [11]. Briefly, fine powder of seeds was made in a blender. Defatted the seed powder of *Silybum marianum *in n-hexane 2-3 times and extracted with aqueous acetone. The extract was concentrated to remove acetone, and then washed by hexane again to remove hydrophobic impurities. The remaining concentrate was treated with 1% NaCl solution to remove water soluble impurities. The precipitate and solid obtained through drying were combined together to form crude Silymarin. The crude SM was washed with aqueous ethanol and then dried completely to give the refined yellowish powder of SM.

### Stock solution preparation

50 mg of SM was suspended in one ml of Dimethyl sulfoxide (DMSO) ensuring stock concentration of 50 μg/μl. Sieving the above solution by using 0.22 um filter inside Laminar Flow Hood, storing at -20°C.

### Cell line

The Huh-7 cell line was offered by Dr. Zafar Nawaz (Biochemistry and Molecular Biology Department, University of Miami, USA). Huh-7 cells were cultured in Dulbecco's modified Eagle medium (DMEM) supplemented with 10% fetal bovine serum & 100 IU/ml penicillin & 100 μg/ml streptomycin, at 37°C in an atmosphere of 5% CO_2_.

### Plasmid construction

For the construction of expression plasmid, viral RNA was extracted from 100 μl serum aliquots using Gentra RNA isolation kit (Gentra System Pennsylvania, USA) according to the manufacturer's instructions. About 200 ng RNA was used for RT-PCR using the SuperScript III one-step RT-PCR system (Invitrogen Life technologies, USA). HCV complementary DNA (cDNA) encoding the full length Core protein (amino acid 1-191 of HCV-3a) were amplified and cloned into pCR3.1 mammalian expression plasmid (kindly provided by Dr. Zafar Nawaz, University of Miami, USA) with FlagTAG inserted at the 5' end of the Core gene with EcoRV and XbaI restriction sites.

### MTT assay for toxicity

To investigate cellular toxicity, 2×10^4 ^cells/well was plated into 96-well plates. After 24 h, different concentrations of SM were added and the plate was sealed and kept at 37°C in an atmosphere of 5% CO^2 ^for 24 h. After the herbal extracts treatment was over, removed the media and SM. 100 μl fresh media and 20 μl of MTT solution (5 mg/ml in PBS) were added to all wells in Columns 1-11. Wrapped the plate in aluminum foil and incubated for 3-4 h at 37°C. Media was carefully removed and added 100 μl of DMSO to dissolve the formazan crystals in Columns 1-11. MTT formazan product was determined by measuring absorbance with an enzyme-linked immunosorbent assay (ELISA) plate reader at a test wavelength of 570 nm and a reference wavelength of 620 nm.

Cell viability was obtained using the following equation:

### Antiviral activity of SM and its fractions against HCV 3a core gene

For transfection studies, Huh-7 cells (5×10^4^) were plated in 24-well plates for 24 h. The medium was removed and cells were washed with 1X PBS. Cells were transiently transfected with expression plasmids containing HCV 3a core gene (0.4 μg) in the presence and absence of SM, its fractions and interferon by using Lipofectamine™ 2000 (Invitrogen life technologies, Carlsbad, CA) according to the manufacturer's protocol. Total RNA was extracted by using Trizol reagent (Invitrogen life technologies, Carlsbad, CA) according to the manufacturer's protocol. To analyze the effect of SM against HCV 3a core gene, cDNA was synthesized with 1 μg of RNA, using Revert Aid TM First Strand cDNA Synthesis Kit (Fermentas, St. Leon-Rot/Germany). Gene expression analysis was carried out via PCR (Applied Biosystems Inc, USA) by using 2X PCR Mix (Fermentas). Following primers were used for the amplification of HCV Core forward primer: GGACGACGATGACAAGGACT; HCV core reverse: GGCTGTGACCGTTCAGAAGT; GAPDH Forward: ACCACAGTCCATGCCATCAC: and GAPDH reverse; TCCACCACCCTGTTGCTGTA PCR was performed by initial denaturation at 95°C for 5 min followed by 30 cycles, each of denaturation at 92°C for 45 s, annealing at 58°C for 45 s, and extension at 72°C for 1 min, with final extension at 72°C for 10 min. The amplified DNA samples were analyzed on 2% agarose gel. The DNA bands were visualized directly under the UV and the photographs of the gels were obtained with gel documentation system.

### Western Blotting

To determine the protein expression levels of HCV 3a core, the transfected and non-transfected cells were lysed with ProteoJET mammalian cell lysis reagent (Fermentas, Canada). Equal amounts of total protein were subjected to electrophoresis on 12% SDS-PAGE and electrophoretically transferred to a nitrocellulose membrane following the manufacturer's protocol (Bio-Rad, CA). After blocking non-specific binding sites with 5% skimmed milk, blots were incubated with primary monoclonal antibodies specific to HCV Core and GAPDH (Santa Cruz Biotechnology Inc, USA) and secondary Horseradish peroxidase-conjugated anti-goat anti-mouse antibody (Sigma Aldrich, USA). The protein expressions were evaluated using chemiluminescence's detection kit (Sigma Aldrich, USA).

### Purification of SM fractions

Purification of SM fractions was done through thin layer chromatography. Briefly, 1% sample solution was prepared in 5 ml solvent and was filtered. Took a TLC card and cut it in 10 cm length and 5 cm width. Small spot of sample was marked on plate. Dried and placed it in chromatography tank having ethyl acetate: chloroform (60: 40) as mobile phase and allowed it to run for one and a half hour. Then, solvent front was marked and chromatogram was dried. TLC chromatogram was observed under UV lamp at wavelength of 254 nm and 366 nm. Separation of components was good and spots of different colors were observed. Pattern of spots was recorded and **R_f _**value of each spot was determined.

## Results

### Toxicological analysis of SM

**Figure 1:**
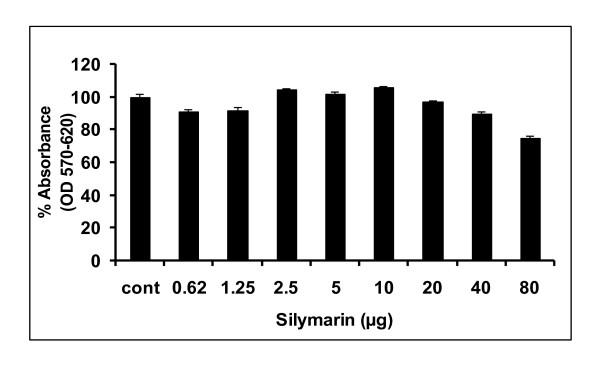
**Toxicity of Silymarin through MTT cell proliferation assay**. Huh-7 cells were plated at the density of 2 × 10^4 ^in 96 well plates. After 24 h cells were treated with different concentrations of SM and control consisted of solvent in which compound dissolved. After 24 h incubation period add MTT solution to all wells and incubated for 3-4 h at 37°C. Viable cells convert MTT to purple formazan crystal. Added DMSO to dissolve the formazan crystals and read absorbance at 570 nm and 620 nm.

Before starting the antiviral screening against HCV, toxicological effect of SM was determined through MTT cell proliferation assay. The MTT substance is reduced by mitochondrial succinic dehydrogenases in living cells to purple formazan crystals that are not soluble in aqueous water. The absorption of dissolved formazan in the visible region correlates with the number of alive cells [12]. Figure 1 shows cytotoxicity analysis of SM at different doses and demonstrates that Huh7 cells viability is unaffected by concentrations up to 20 μg. However, when the concentration exceeded 40 to 80 μg, toxic effect in liver cells were observed. The data was verified by microscopic examination of cells and standard trypan blue dye measurement, which demonstrates that SM has no toxic effect at a concentration of 20 μg.

### Antiviral effect of SM against HCV 3a Core gene

**Figure 2:**
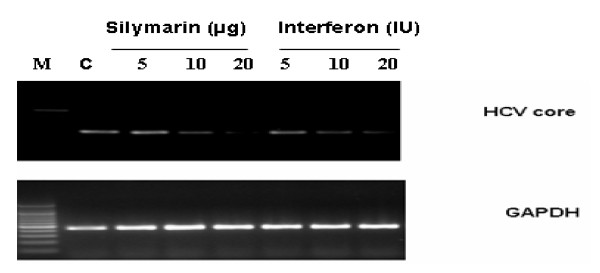
**Antiviral effect of SM against HCV core gene in Liver cells**. Huh-7 cells were transfected with Core in the presence and absence of different concentration of SM and interferon. After 24 h incubation period, total RNA was extracted and the levels of HCV core gene were determined by RT-PCR.

To determine the antiviral effect against HCV core gene, Huh-7 cells were transfected with HCV core gene in the presence and absence of different concentrations of SM and interferon. After 24 h, RNA was extracted through Triazol (Invitrogen). cDNA were generated by oligo dT primer. cDNA was amplified by PCR using primers specific to the HCV core gene of 3a genotype. Amplification of GAPDH mRNA served as an internal control. Figure 2 demonstrates that SM inhibits HCV RNA expression in a dose-dependent manner, while GAPDH mRNA expression remains unaffected by the addition of the compound. Moreover, SM inhibits HCV core expression or function similar to interferon. This may be due to direct effect of SM against HCV 3a core or activation of JAK/STAT pathway.

### Purification of different fractions of SM by thin layer chromatography

**Figure 3:**
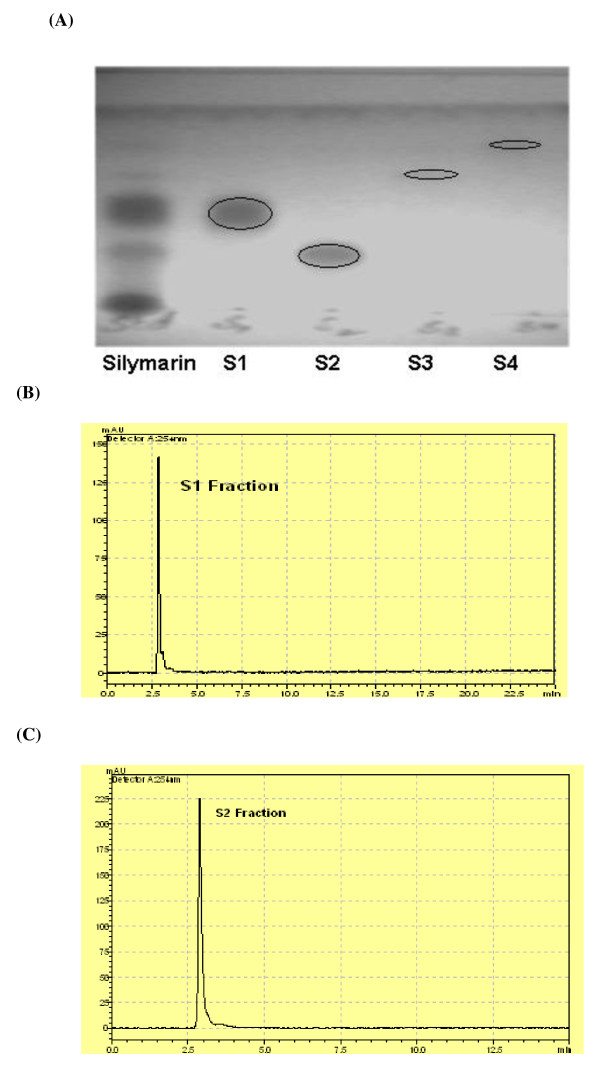
**TLC and HPLC chromatogram of SM Fractions**. (A) Ran SM extract in TLC plate. Analyzed the sample at UV light of 254 wavelength. It separates into four components S1, S2, S3, and S4 in solvent (ethyl acetate: chloroform 60:40) with Rf values 0.51, 0.40, 0.64 and 0.75 respectively. (B) HPLC Chromatogram of S1 fraction. (C) HPLC chromatogram of S2 fraction.

**Figure 4:**
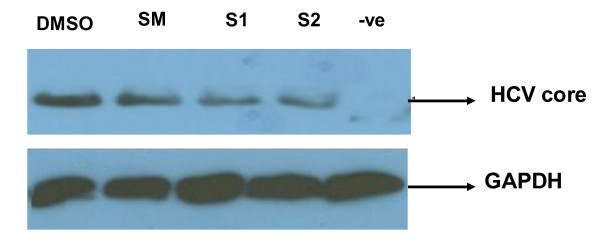
**HCV core gene inhibition by SM and its Fractions S1 and S2**. Huh-7 cells were transfected with core in the presence and absence of 10 μg SM and its fractions SI and S2. After 48 h incubation period, protein was isolated and analyzed by western blotting with anti -Core monoclonal antibody and GAPDH served as internal control.

Crude extract of SM was fractioned by thin layer chromatography (TLC). SM separates in to four components S1, S2, S3 and S4 in solvent (ethyl acetate: chloroform) with Rf value 0.51, 0.40. 0.64 and 0.75 respectively. Purity of each fraction was checked by HPLC (Figure 3). For large scale purification packed the column with silica and dried the sample by mixing the silica. Allowed the solvent (ethyl acetate: chloroform 60: 40) to flow down with 1 ml/min. Fractions were collected after half an hour. Then fractions were combined after checking each fraction in TLC plate at 254 wavelength to obtain purely separate components (S1, S2, S3, and S4). The individual fraction was then solubilized in DMSO, and tested for antiviral screening against HCV. Western blotting results showed a dramatic reduction at the protein level of HCV core protein in cells treated with SM and its fractions S1 and S2; whereas the expression levels of GAPDH protein remained the same in control verses treated cells (Figure 4).

## Discussion

HCV infection is a serious global health problem that affects 180 million people worldwide and 10 million people in Pakistan. Currently, no vaccine is available for prevention of HCV infection due to high degree of strain variation. The current treatment with PEG-INFα in combination with ribavirin is costly, has significant side effects and fail to cure about one half of all infections [13,14]. Hence, there is a need to develop other anti-HCV agents, which are less toxic, more efficacious and cost-effective. It is well known that medicinal plants have been used for centuries against different diseases including viral diseases and were therefore, used during the last century to identify, isolate and purify new compounds to treat viral and bacterial diseases. Many traditional medicinal plants and herbs were reported to have strong antiviral activity against DNA and RNA viruses by inhibiting virus replication, interfering with virus-to-cell binding and immunomodulation actions [15,16]. HCV structural proteins (core, E1 and E2) and nonstructural proteins (NS3 protease and NS5B RNA-dependent RNA polymerase) are potent molecular targets of new antiviral compounds.

*Silybum marianum *is a flavolignin commonly known as 'milk thistle'. *Silybum marianum *has been shown to have clinical applications in the treatment of toxic hepatitis, fatty liver, cirrhosis, ischemic injury, radiation toxicity, and viral hepatitis. SM has been found to suppress nuclear factor kappa B (NF kB) dependent gene expression and also has inhibitory action on inflammatory and cytotoxic cascade of events induced by the viral infection (Saller et al., 2001). SM has anti-oxidative effect by inhibiting free radicals such as superoxide radical, hydroxyl radical (OH), hydrogen peroxide (H_2_O_2_), and lipid peroxide radicals [17]. SM has been shown to inhibit the growth of endothelial [18], lung tumor [19], prostate cancer [20], and human hepatoma HepG2 and Hep3B cells. The anti-proliferative actions of SM are due to inhibition of signaling pathways that regulate the cell cycle including Akt and cyclin-dependent kinases. SM is a complex mixture of four flavonolignan isomers, namely silybin, isosilybin, silydianin and silychristin with an empirical formula C_25_H_22_O_10. _Silybin is the major and most active component and represents about 60-70 percent, followed by silychristin (20%), silydianin (10%), and isosilybin (5%) [21]. SM plays an important part in modulating cell membrane and membrane receptors by inhibiting the binding of epidermal growth factor [22]. Thus, SM shows hepatoprotective effect by alterations in the hepatic membrane. However, the results, of different clinical trials show that SM reduces liver enzymes such as ALT and AST associated with hepatitis [23]. Treatment of 50 HCV patients with a mix of 7 antioxidants including SM also reduced ALT levels in 44% of patients, and reduced viral load in 25% of patients [24]. SM has anti-proliferative properties due to the modulation of specific signaling pathways, transcription factors and gene expression [25]. SM also effect on hepatic stellate cells (HSC) and decreases HCV-induced fibrosis [26]. A recently published clinical trial, shows that high doses of intravenous silibinin results potent antiviral activity against HCV infected patient [27].

HCV Core protein modulates gene transcription, cell proliferation, cell death and cell signaling, interferes with metabolic genes and suppresses host immune response [28] leading to oxidative stress, liver steatosis and eventually hepatocellular carcinoma [29]. Core protein is also able to up-regulate cyclooxygenase-2 (Cox-2) expression in hepatocytes derived cells, providing a potential mechanism for oxidative stress [30]. The expression of Cox-2 in HCC was found to correlate with the levels of several key molecules implicated in carcinogenesis such as inducible nitric oxide synthetase (iNOS), activate vascular endothelial growth factor (VEGF) and phosphorylated Akt (p-Akt) [31,32]. In this study, SM was examined for toxicological analysis in Huh-7 line. Our data show that SM is non toxic at a concentration up to 20 μg but when exceed from 30 μg SM show toxic effect in both liver and fibroblast cells (Figure 1). The study of Polyak et al also agreed with the statement that SM has non toxic up to 20 μg concentration [11]. After toxicological analysis, antiviral effect against HCV core gene of 3a genotype was analyzed and our results showed that SM resulted in dose-dependent inhibition of HCV core gene similar to interferon alpha 2a (Figure 2). This may be due to stimulation of interferon pathway by phosphorylation of Stat1 on tyrosine and serine [11]. SM may have effective on hepatocyte membranes, interferon receptor, inhibit of negative regulators of the Jak-Stat pathway such as SH2-containing phosphatases, the protein inhibitors of activated STATs, or the suppressors of cytokine signaling proteins such as SOCS 1 and SOCS 3 proteins [33]. Studies in cell culture models have suggested a role for SOCS3 in HCV IFN resistance. HCV core protein over-expression has been reported to induce SOCS3 in cell culture models, resulting in impaired IFN signaling. HCV replicon cells resistant to IFN therapy have been shown to produce higher levels of SOCS3; inhibition of SOCS3 expression results in production of interferon [34]. Furthermore, several *in-vivo *studies have shown that hepatic SOCS3 expression is associated with response to IFN treatment. Other possible mechanism could involve such as IRF3 or IRF9 and MAPK pathways [35]. In order to identify the active ingredient against HCV, SM was fractioned by thin layer chromatography (TLC), column chromatography and HPLC. Two fractions (S1 and S2) out of four showed antiviral effect against HCV core gene expression or function. These results showed that SM contains potential phytochemicals such as S1 and S2 and combination of these agents with interferon is helpful to cure HCV infection.

## Abbreviations

**HCV**: Hepatitis C virus; **SM: **Silymarin; **Huh-7**: Human Hepatoma Cell line.

## Competing interests

The authors declare that they have no competing interests.

## Authors' contributions

UAA contributed in lab work and manuscript writes up. TJ and SDR help me in chromatographic techniques. SRD and ZN was the principal investigator and provide all facilities to complete this work. All the authors read and approved the final manuscript.

## Authors' information

Usman Ali Ashfaq (PhD Molecular Biology), Tariq Javed (M.Phil pharmaceutical chemistry, Sidra Rehman (MSc Chemistry) and Sheikh Riazuddin (PhD molecular Biology and Dean Post graduate study at Allama Iqbal medical college, Lahore.
